# Palliation of malignant dysphagia with a segmented self-expanding metal stent: A STROBE-compliant article

**DOI:** 10.1097/MD.0000000000027052

**Published:** 2021-08-27

**Authors:** Marie-Sophie Wiese, Thomas Dratsch, Patrick Sven Plum, Florian Lorenz, Isabel Rieck, Daniel Pinto dos Santos, Hakan Alakus, Marc Bludau, Robert Kleinert, Tobias Goeser, Christiane Josephine Bruns, Seung-Hun Chon

**Affiliations:** a University of Cologne, Cologne, Germany; b Department of Radiology, University of Cologne, Faculty of Medicine, and University Hospital Cologne, Cologne, Germany; c Department of General, Visceral, Cancer and Transplantation Surgery, University of Cologne, Faculty of Medicine, and University Hospital Cologne, Cologne, Germany; d Department of Gastroenterology and Hepatology, University of Cologne, Faculty of Medicine, and University Hospital Cologne, Cologne, Germany.

**Keywords:** dysphagia, esophageal adenocarcinoma, malignant tumors of the esophagus, palliation, self-expanding segmented stent, stent

## Abstract

Self-expanding metal stents (SEMSs) in different geometric shapes are an established palliative treatment for malignant tumors of the esophagus. Mechanical properties and stent design have an impact on patient comfort, migration rate, and removability. SEMS with a segmented design (segSEMS) have recently become available on the market, promising new biomechanical properties for stent placement in benign and malignant esophageal diseases. In this study, we evaluated recurrent dysphagia, quality of life as well as technical success and complications for segmented SEMS-implantation in a retrospective study in palliative patients with dysphagia caused by malignant tumors of the esophagus.

Between May 2017 and December 2018, patients presented to the interdisciplinary department of endoscopy of the University Hospital Cologne underwent segmented SEMS placement for malignant dysphagia. Patient follow-up was evaluated, and complications were monitored. Quality of life and functional improvement were monitored using the EORTC QLQ-C30 and QLQ-OE18.

A total of 20 consecutive patients (16 men, 4 women; mean age: 65.5, range: 46–82) participated in the study and were treated with 20 segSEMS in total. The success rate of stent placement was 100%. Stent migration occurred in 3 patients (15.0%). Insertion of segSEMS immediately lead to a 48.0% reduction of dysphagia in the first 2 months (*P* < .001). Pain while eating (odynophagia) could also be significantly reduced by 39.6% over the first 2 months (*P* < .001).

Implantation of segSEMS is a feasible and effective treatment for dysphagia in palliative patients with malignant tumors of the esophagus, offering immediate relief of symptoms and gain of physical functions.

## 1 Introduction

Palliation of the main complaints of patients with esophageal cancer, such as dysphagia, plays an important role in the management of incurable malignant tumors of the esophagus.^[1–3]^ In current clinical practice, esophageal stents are mostly used for palliation of dysphagia.^[4–6]^ Even though stents can successfully reduce dysphagia, some of the common problems are migration, occlusion, and bleedings.^[7]^ Several innovations, such as self-expandable metallic and plastic stents, different methods to cover the stent (fully covered vs partially covered vs uncovered) as well as drug eluting or radioactive stents have been introduced over the years to mitigate those problems.^[8–11]^ Recently, segmented self-expanding metal stents (segSEMS), in which individual elements are connected by a nylon wire, have become available. Due to their segmented nature and ability to bend, they promise to reduce pressure on the esophagus, offer a better fit over curved surface areas, and better adjust to esophageal peristalsis, which may result in less complications. In a recent in-vitro evaluation of the mechanical properties of segSEMS, it was shown that segSEMSs with a high radial, high local radial, and a low axial force may be a better option for the conditions in the lumen of a cancerous esophagus, causing less pressure on the esophageal wall.^[12]^ This may result in less migration and higher patient comfort. First studies have shown the safety and feasibility of implanting segmented stents.^[13–15]^ However, it has not yet been demonstrated whether segSEMSs also improve the quality of life in patients with incurable malignant tumors of the esophagus.

Therefore, the main goal of the present study was to evaluate the implantation and feasibility of a new segSEMS in a group of palliative patients with malignant tumors of the esophagus, focusing on quality of life using standardized instruments (EORTC QLQ-C30) as well as functional improvement questionnaires (QLQ-OE18) (eg, dysphagia).^[16,17]^

## 2 Methods

### 2.1 Study design

This retrospective study was performed at the Department of General, Visceral, Cancer, and Transplantation Surgery at the University Hospital Cologne, which is a national referral center for surgery of the upper gastrointestinal tract. The segSEMS (ESO, Endo-flex GmbH, Voerde, Germany) was used in 20 consecutive patients who were treated at our hospital between May 2017 and December 2018 for malignant dysphagia. Patients with the following histopathological subtypes were included: esophageal adenocarcinoma (EAC), esophageal squamous cell carcinoma (ESCC), and neuroendocrine tumors (NET). Technical success, complications, patient follow-up, and quality of life were monitored and evaluated for 6 months or until the death of a patient. Mean time of follow-up was 159 days (standard deviation = 74 days). The analysis was performed retrospectively.

### 2.2 Statement of ethics

Ethics Committee approval for this retrospective study was obtained before the study (Ethics Committee, University of Cologne) and adheres to the criteria of the ethics committee of the University of Cologne (No. 17–207). Written informed consent was given by all patients before study inclusion.

### 2.3 Patient selection

Between May 2017 and December 2018, 20 consecutive patients (16 men, 4 women; mean age: 65.5, range: 46–82) were selected to be treated with a segSEMS (see Table 1). All patients were discussed in our internal tumor board and were classified as inoperable. Hence, palliative treatment was suggested. To provide the best supportive care, they were recommended to be treated with a segSEMS. The patients had to meet 1 or a combination of the following criteria to be included in the study: significant dysphagia caused by a histologically proven, malignant tumor of the esophagus (EAC, ESCC, or NET), inoperability due to lack in general condition, unresectability because of tumor extent, metastases, or involvement of the lymph nodes. The following exclusion criteria were applied: patient refusal, other already scheduled therapies or surgery, and lack of study supervisor.

**Table 1:** Demographic data of included patients.

Variable	Values
Mean age (range), yr	45.5 (42–82)
Men/women	16/4
Median ASA-score (range)	3 (3–4)
Mean weight (range), kg	68.1 (42–110)
BMI (range)	22.6 (16.4–32.1)
Histology	
Adenocarcinoma	13 (65.0%)
Neuroendocrine tumor	1 (5.0%)
Squamous cell carcinoma	6 (30.0%)
Stage	
T1 Nx	1 (5.0%)
T2 Nx	2 (10.0%)
T2 N+	1 (5.0%)
T3 Nx	1 (5.0%)
T3 N+	15 (75.0%)
Location of tumor	
Upper	0 (0.0%)
Middle	8 (40.0%)
Lower	12 (60.0%)
Mean tumor length (range), cm	5.4 (2–8)
Additional cancer therapy	
None	5 (25.0%)
Chemotherapy	3 (15.0%)
Radiotherapy	4 (20.0%)
Both	8 (40.0%)

ASA = American Society of Anaesthesiologists Classification, BMI = body mass index.

### 2.4 Quality of life (EORTC QLQ-C30) and esophageal cancer module (QLQ-OE18)

Quality of life was assessed using the quality of life scale of the German version of the EORTC QLQ-C30.^[16]^ The EORTC QLQ-C30 is a cancer specific, multidimensional questionnaire developed by the European Organisation for Research and Treatment of Cancer, which has been internationally field tested and is applicable across a range of cultural settings. To evaluate symptoms and function, the German version of the esophageal cancer module QLQ-OE18 was used.^[17]^ The QLQ-OE18 is a questionnaire that has been specifically developed and validated to assess symptoms and functions in patients with esophageal cancer and complements the EORTC QLQ-C30. The QLQ-OE18 consists of 18 questions measuring dysphagia as well as 9 symptoms (eating, reflux, pain, trouble swallowing saliva, choked when swallowing, dry mouth, trouble with taste, trouble with coughing, and trouble talking). The questions are rated on a scale ranging from 1 (not at all) to 4 (very much). Dysphagia was measured using 3 items (Could you eat solid foods?, Could you eat liquidised or soft foods?, and Could you drink liquids?). Pain was also measured using 3 items (Have you had pain when you eat?, Have you had pain in your chest?, and Have you had pain in your stomach?). The assessment was carried out before the stent implantation, 1 week after implantation and then in monthly intervals. Follow-ups were carried out for 6 months. Furthermore, all complications, such as bleeding or migration, were monitored and all endoscopic re-interventions recorded.

### 2.5 Stent patency

Stent patency has been identified as an important factor in the evaluation of stents.^[18]^ In our study, stent patency was assessed as part of the QLQ-OE18 before stent implantation, 1 week after implantation and then in monthly intervals.

### 2.6 Segmented SEMS description and procedure

**Figure 1:**
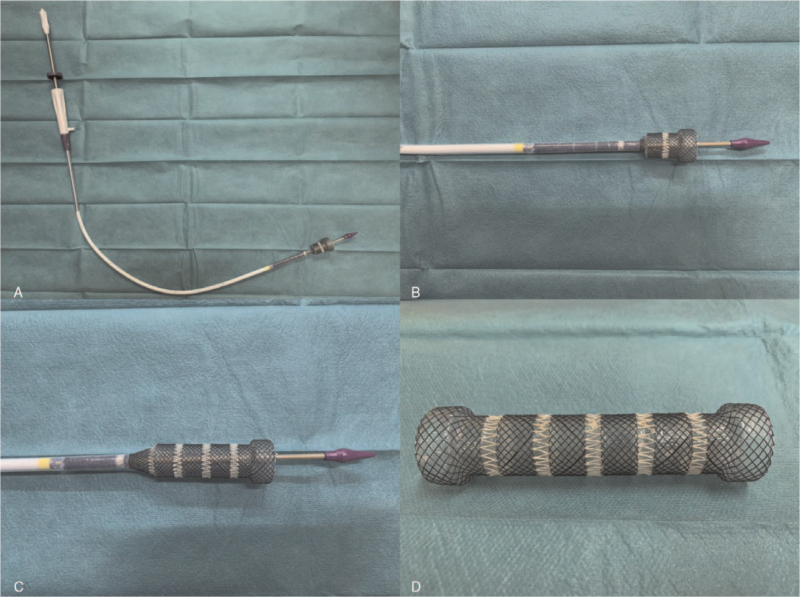
segSEMS (Endo-flex GmbH, Germany) used in the study. A) Delivery system, catheter, and outer tube, B) + C) release of the segSEMS, and D) released and expanded segSEMS. segSEMS = segmented self-expanding metal stent.

The esophageal segSEMS (Endo-flex GmbH, Germany) (shown in Fig. 1) is made of braided nitinol, a nickel–titanium alloy, and is fully covered in silicone to prevent tissue ingrowth. The flare end segments have a diameter of 30 mm and a length of 15 mm. They are connected by 4 nitinol elements with a diameter of 20 mm and an individual length of 10 mm. Each segment is connected with a nylon wire with a length of 5 mm. The stent consists of a delivery system loaded with a fully covered, segSEMS, which is mounted on an inner catheter and constrained by an outer tube. The delivery system has a length of 700 mm and a diameter of 8 mm (24 F). The segSEMS is released distally by retracting the outer tube.

### 2.7 Stent treatment

All procedures in this study were performed by 2 experienced endoscopists. The procedures were performed under sedation with PROPOFOL (Fresenius Kabi, Bad Homburg, Germany). For endoscopic guidance throughout the stent placement, a flexible video esophagogastroduodenoscope (GIF-H190; Olympus Medical Systems, Tokyo, Japan) was used. After assessing the size and height of the tumor, stent position was determined. After advancing the delivery system to the constriction over the guidewire, the segSEMS was carefully deployed under endoscopic guidance and sufficient expansion was verified. In case of stent failure and to ensure patient safety, conventional stents were implanted.

### 2.8 Statistics

The data were analyzed using SPSS Version 26 (IBM SPSS Statistics, Version 26.0.0.0 64-Bit-Version, IBM Corp. USA). Demographic data were summarized using descriptive statistics. Continuous variables are summarized as means and 95% confidence intervals are reported. Repeated measures-ANOVAS were performed to analyze the development of the QLQ-C30 and QLQ-OE18 over time. *P* < .05 was considered statistically significant.

## 3 Results

### 3.1 Endoscopic results

A total of 20 segSEMSs were successfully inserted in all 20 patients. There were no technical complications (0.0%) and none of the patients died during the procedure (0.0%) leading to a technical success rate of 100.0%. Over the course of the study, our overall complication rate was 30% (6 out of 20 cases). On average, complications occurred 72 days after stent placement (range: 28–110). Stent migration occurred in 3 patients (15.0%). Of the 3 stents that migrated, 2 were originally deployed in the middle of the esophagus at 27 and 29 cm and 1 was deployed in the lower esophagus at 33 cm. In 2 of those cases, the stent was endoscopically repositioned. In 1 case, the stent could be removed due to regression of tumor growth. Additionally, in 3 cases (15.0%) stents had to be renewed due to bleeding (1 patient; 5.0%), tumor ingrowth (1 patient; 5.0%), and a fistula (1 patient; 5.0%). In all 3 cases, the segmented stents were replaced with SEMSs.

### 3.2 Stent patency

**Figure 2:**
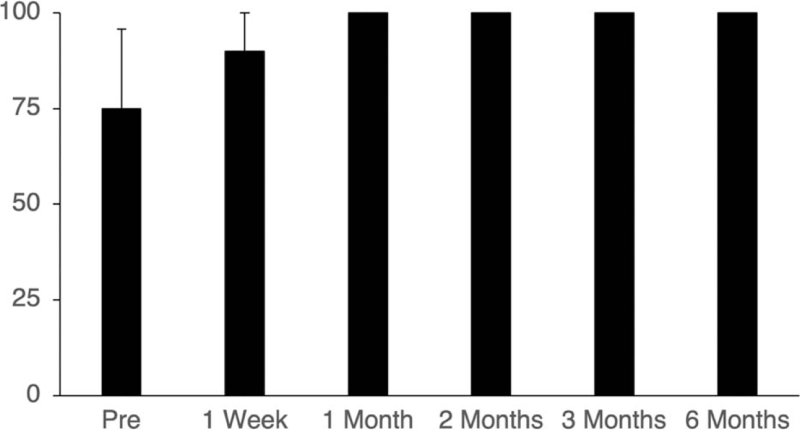
Stent patency over 6 months. Error bars represent 95% confidence intervals.

Figure 2 shows the results for stent patency. Before stent placement, 75% of patients (15 out of 20) were able to eat soft food and drink liquids. One week after stent placement, 90% of patients (18 out of 20) were able to eat soft food and drink. One month after stent placement, all patients (100%, 20 out of 20) were able to eat soft food and drink liquids. Stent patency remained high until the end of the study period. At the end of the study period, all of the remaining 7 patients were still able to eat soft food and drink.

### 3.3 Survival

**Figure 3:**
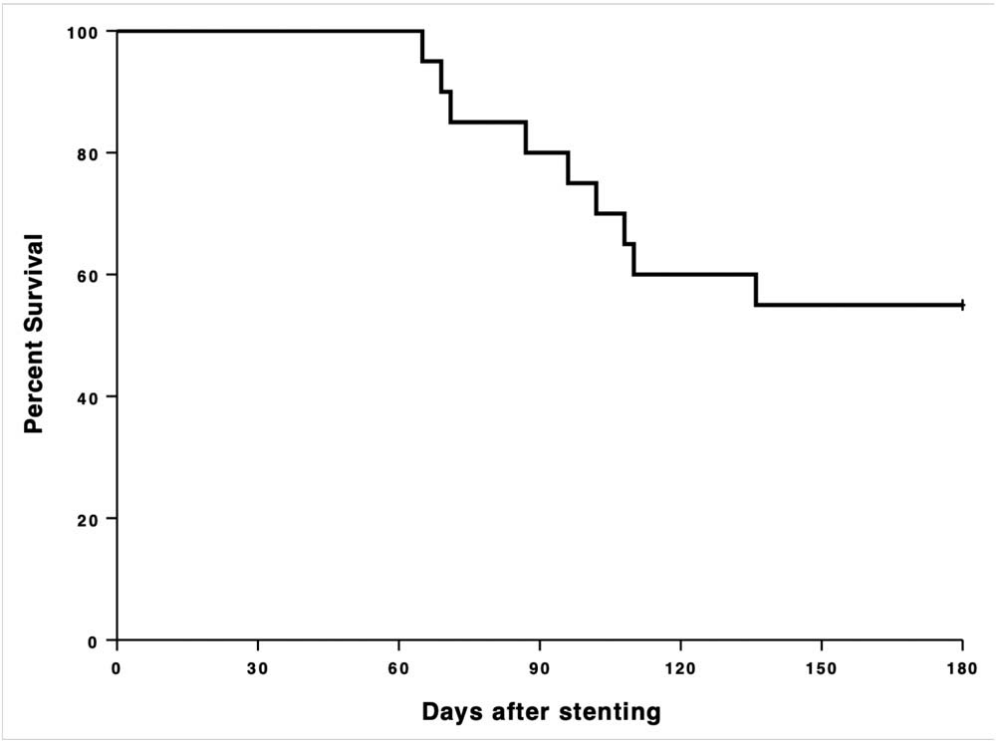
Percent survival until follow-up after 6 months (180 days).

At the six-month follow-up, 11 out of 20 patients (55.0%) were still alive (see Fig. 3). For the patients who died before the six-month follow-up, the median survival after stent implantation was 96 days (range: 65–136 days).

### 3.4 Quality of life (EORTC QLQ-C30) and esophageal cancer module (QLQ-OE18)

**Figure 4:**
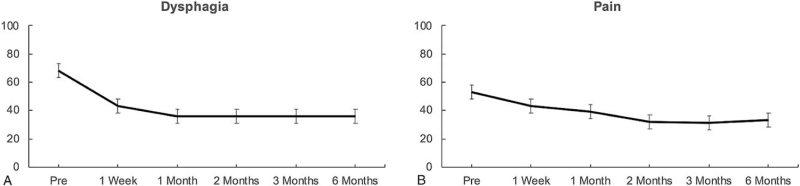
Mean values and 95% confidence intervals for A) dysphagia and B) pain while eating over the course of the study.

Tables 2 and 3 show the descriptive and statistical results for quality of life and the functional scales over the course of the study. As was expected in palliative patients with malignant tumors of the esophagus, quality of life did decrease significantly over the course of 6 months (*P* = .02). With regard to functional improvement, as Figure 4 shows, insertion of segSEMS immediately lead to a 48.0% reduction of dysphagia in the first 2 months (*P* < .001). At the 6 months follow-up, a significant 32.2% reduction of dysphagia was still measurable (*P* < .001). Pain while eating (odynophagia) could also be significantly reduced by 39.6% over the first 2 months (*P* < .001) (see Fig. 4). Chocking when swallowing was also reduced by 52.2% in the first 2 months (*P* = .01). Difficulties while eating were also significantly reduced in the first 2 months by 16.3% (*P* = .02). However, reflux symptoms did increase over the course of 6 months (*P* < .001). Trouble with coughing did decrease after stent implantation, but did rise again to baseline levels after 2 months (*P* = .04). All other subscales of the QLQ-OE18 were not significant.

**Table 2:** Mean values (95% confidence intervals) for the quality of life scale of the QLQ-C30 and the QLQ-OE18 over the course of the study.

	Pre (N = 20)	1 week (N = 20)	1 month (N = 20)	2 months (N = 20)	3 months (N = 15)	6 months (N = 7)
QLQ-C30						
Global health status	35 (24–46)	35 (28–42)	38 (33–44)	33 (27–40)	33 (23–43)	13 (3–23)
QLQ-OE18						
Dysphagia	68 (61–76)	43 (32–55)	36 (27–44)	36 (27–44)	36 (23–48)	46 (39–53)
Pain	53 (36–71)	43 (30–56)	39 (29–50)	32 (23–41)	31 (19–43)	33 (8–59)
Choked when swallowing	38 (22–55)	25 (14–36)	23 (13–34)	18 (9–28)	20 (6–34)	14 (0–31)
Eating	77 (65–88)	71 (61–82)	68 (57–79)	64 (55–73)	68 (55–81)	80 (76–84)
Reflux	34 (22–47)	47 (34–59)	53 (41–66)	55 (41–69)	60 (42–78)	67 (51–82)
Trouble swallowing saliva	50 (32–68)	33 (21–46)	35 (22–48)	32 (20–44)	27 (16–37)	38 (17–59)
Dry mouth	62 (47–76)	57 (40–74)	50 (34–66)	50 (34–66)	56 (32–79)	81 (57–100)
Trouble with taste	43 (28–59)	37 (22–51)	47 (30–63)	52 (35–68)	56 (36–75)	71 (60–83)
Trouble with coughing	47 (31–62)	32 (19–45)	33 (22–45)	43 (29–58)	40 (24–56)	33 (16–51)
Trouble talking	22 (7–36)	25 (13–37)	28 (17–40)	25 (13–37)	24 (8–41)	14 (0–39)

**Table 3:** Results of the repeated-measures ANOVAs for the quality of life scale of the QLQ-C30 and the QLQ-OE18.

	2 months			3 months			6 months		
	F	*P*	pEta^2^	F	*P*	pEta^2^	F	*P*	pEta^2^
QLQ-C30									
Global health status	.491	.690	.025	.585	.674	.040	**2.66**	**.022** ^∗^	**.307**
QLQ-OE18									
Dysphagia	**20.72**	**.001** ^∗∗^	**.522**	**12.91**	**.001** ^∗∗^	**.480**	**8.75**	**.001** ^∗^	**.593**
Pain	**7.41**	**.001** ^∗∗^	**.281**	**5.04**	**.002** ^∗∗^	**.265**	1.94	.088	.244
Choked when swallowing	**4.09**	**.011** ^∗^	**.177**	**2.81**	**.034** ^∗^	**.167**	0.35	.926	.055
Eating	**3.49**	**.021** ^∗^	**.155**	0.93	.456	.062	2.20	.054	.268
Reflux	**4.09**	**.011** ^∗^	**.177**	**3.19**	**.020** ^∗^	**.185**	**5.34**	**.001** ^∗∗^	**.471**
Trouble swallowing saliva	2.47	.071	.115	1.47	.225	.095	0.33	.934	.053
Dry mouth	1.35	.268	.066	1.34	.268	.087	1.99	.080	.249
Trouble with taste	1.80	.158	.086	1.63	.179	.104	1.36	.249	.184
Trouble with Coughing	**2.82**	**.047** ^∗^	**.129**	2.13	.089	.132	1.25	.298	.172
Trouble talking	0.40	.755	.021	0.06	.993	.004	0.46	.861	.071

pEta^2^ = partial Eta^2^.

∗ *P* < .05.

∗∗ *P* < .001.

## 4 Discussion

In a palliative setting, the main aim of therapy is to maintain or increase quality of life and physical functioning. One important aspect of quality of life is the biological, social, and–most importantly–pain-free intake of food and drinks. In advanced stage malignant tumors of the esophagus, food-intake is often severely limited due to tumorous stenosis of the esophagus. Therefore, the implantation of a stent offers immediate relief of symptoms and can significantly improve quality of life. Thus, the main goal of the present study was to analyze the insertion of an innovative segSEMS in a cohort of palliative patients with malignant tumors of the esophagus (EAC, ESCC, and NET), focusing on quality of life and functional improvement. Even though overall quality of life did decrease due to progression of disease, as also shown in another study,^[19]^ insertion of segSEMS did lead to an immediate improvement of dysphagia by 48.0% in the current analysis. Additionally, stent patency was 100.0% 1 month after the intervention and remained 100.0% until the end of the study. Pain while eating (odynophagia) was also improved by 39.6%, and choking when swallowing was improved by 52.2%. Difficulties while eating, as measured in the QLQ-OE18, could also be reduced by 16.0%. Especially the reduction in pain might be attributed to the segmented nature of the stent, allowing an individual, pressure-reduced fit over a variety of irregular tumorous surfaces and peristaltic movement.

Comparing our results to a recent study on the feasibility of segSEMSs, we observed a lower rate of complications in our study of 30.0% (6 out of 20) compared to 45.8% (11 out of 24).^[14]^ Additionally, we could demonstrate a lower rate of migration of 15.0% (3 out of 20) in our data compared to 37.5% in the previous published work (9 out of 24).^[14]^ One possible explanation for this might be the different stents used in our study (ESO, Endo-flex GmbH, Voerde, Germany) and the study by Bi et al (ST71, Micro-Tech, Nanjing, China). Both stents (ESO and ST71) have an almost identical local axial force and therefore provide axial flexibility and support esophageal motility. One of our main focusses was the reduction of pain in palliative patients with esophageal cancer. Hence, we chose the ESO over the ST71 because it has a slightly lower local radial expansion force and would therefore put less stress on the esophageal wall and cause less pain. Comparing our results with the results from Bi et al^[14,15]^ that used the ST71, the lower local radial expansion force of the ESO did not result in more migrations in our study.

One of the main strengths of our study was the measurement of quality of life and physical functioning by using standardized instruments (EORTC QLQ-C30 and QLQ-OE18), which allowed us to compare the effectiveness of the segSEMS with other therapeutic options, such as conventional stents or brachytherapy.^[20]^

Our study surely has certain limitations: Firstly, as we conducted our study in a palliative setting, we were only able to include a small sample of patients. Even though our results are promising, larger studies are needed to further evaluate the benefits and complication rates of segSEMSs. Secondly, our study did not include a control group that was treated with conventional SEMS. However, comparing our results to recent studies, our migration rate (15.0%) was slightly lower than that of conventional fully-covered stents (20.0%).^[19]^

## 5 Conclusion

The insertion of segSEMSs is a feasible and effective treatment for dysphagia in palliative patients with malignant tumors of the esophagus, offering immediate relief of symptoms and gain of physical functions.

## Acknowledgments

None.

## Author contributions

**Conceptualization:** Marie-Sophie Wiese, Seung-Hun Chon.

**Data curation:** Marie-Sophie Wiese, Thomas Dratsch, Marc Bludau, Christiane J Bruns.

**Formal analysis:** Marie-Sophie Wiese, Thomas Dratsch.

**Investigation:** Patrick Sven Plum, Seung-Hun Chon.

**Methodology:** Marie-Sophie Wiese, Isabel Rieck, Hakan Alakus.

**Project administration:** Marie-Sophie Wiese, Robert Kleinert, Tobias Goeser.

**Resources:** Patrick Sven Plum, Daniel Pinto dos Santos, Seung-Hun Chon.

**Software:** Isabel Rieck.

**Supervision:** Christiane J Bruns, Seung-Hun Chon.

**Validation:** Florian Lorenz, Robert Kleinert, Seung-Hun Chon.

**Visualization:** Daniel Pinto dos Santos, Tobias Goeser.

**Writing – original draft:** Marie-Sophie Wiese, Thomas Dratsch.

**Writing – review & editing:** Marie-Sophie Wiese, Thomas Dratsch, Patrick Plum, Isabel Rieck, Florian Lorenz, Daniel Pinto dos Santos, Hakan Alakus, Marc Bludau, Robert Kleinert, Tobias Goeser, Christiane J Bruns, Seung-Hun Chon.
